# Three missense mutations of DNA topoisomerase I in highly camptothecin-resistant colon cancer cell sublines

**DOI:** 10.3892/or.2013.2594

**Published:** 2013-07-05

**Authors:** YASUHIRO ARAKAWA, KOJI OZAKI, YUTAKA OKAWA, HISASHI YAMADA

**Affiliations:** 1 Department of Oncology and Hematology, Jikei University School of Medicine, Minato-Ku, Tokyo 105-8471, Japan; 2 Department of Molecular Genetics, Jikei University School of Medicine, Minato-Ku, Tokyo 105-8471, Japan; 3 Department of Internal Medicine, Sasaki Hospital, Fukaya, Saitama 366-0824, Japan

**Keywords:** DNA topoisomerase I, camptothecin resistance, indolocarbazole resistance, colorectal cancer

## Abstract

Various anticancer drugs, including camptothecins and indolocarbazoles, target DNA topoisomerase I (Top1). We previously described the camptothecin-resistant colon cancer cell line DLDSNR6, which has a Gly365Ser missense mutation in Top1. In the present study, we established highly camptothecin-resistant sublines from DLDSNR6 cells by continuous exposure to higher camptothecin concentrations. The established sublines grew in the presence of 30 μM of camptothecin, but exhibited markedly retarded growth. In addition to Gly365Ser, these sublines harbored a Top1 Gly717Arg mutation and some had also a Top1 Gln421Arg mutation. Top1 activity was reduced to approximately one-eighth in highly resistant cell lines compared with that in parental DLD-1 cells. Resistant clones with 3 Top1 mutations including Gln421RArg exhibited the highest resistance to the indolocarbazole J-107088 in terms of the effect on the cell cycle distribution. The Gln421 mutation was equivalent to a mutation recently found in camptothecin biosynthesizing plants, but it has not previously been found in mammalian cells.

## Introduction

DNA topoisomerase I (Top1) is an essential enzyme in higher eukaryotes as well as the prime intracellular target of various classes of anticancer drugs, such as camptothecins, indenoisoquinolines and indolocarbazoles (1–3). Top1 catalyzes the relaxation of DNA supercoiling to allow the processes of replication, transcription, and recombination to occur by reversibly nicking one strand and forming transient DNA cleavage complexes (4). Under physiological conditions, cleavage complexes are transient. Top1-targeting drugs, which act as ‘interfacial inhibitors’, stabilize covalent Top1-DNA complexes and cause DNA strand breaks that lead to the apoptosis of drug-treated cells (5).

The underlying mechanisms of the resistance to Top1-targeting drugs may involve the inappropriate accumulation of drug in the tumor cells, mutations in Top1, or changes in the cellular response to DNA strand breaks. Mutations of Top1 that give culture cells resistance to Top1-targeting drugs have been identified (6). We previously established a camptothecin-resistant colon cancer cell line, which was designated DLDSNR6, and identified a missense mutation of the Top1 gene that resulted in a glycine to serine substitution at codon 365. In these resistant cells, Top1 shows lower catalytic activity and camptothecin traps fewer Top1-DNA complexes than parent DLD-1 cells (7).

Camptothecin is a plant alkaloid produced by the Chinese tree *Camptotheca acuminata*. Camptothecin and its derivatives are potent poisons to most eukaryotic cells, including those of higher plants, but camptothecin-producing trees are insensitive to these self-producing toxic metabolites. Sirikantaramas *et al*(8) demonstrated that camptothecin-producing plants have point mutations in the Top1 gene at Asn421, Leu530 and Asn722, which confer resistance to camptothecins. Although Top1 mutations at codon 722 have been identified in several camptothecin-resistant human cancer cell lines, the other mutations have yet to be found (9).

## Materials and methods

### Materials

SN-38 was kindly provided by Yakult Co., Ltd. (Tokyo, Japan), and J-107088 was kindly supplied by MSD K.K. (Tokyo, Japan, formerly Banyu Pharmaceutical Co., Ltd). Other chemicals were purchased from Sigma-Aldrich Japan K.K. (Tokyo, Japan). SN-38, J-107088, camptothecin and Ko143 were resuspended with Me_2_SO as stock solutions and stored at −20ºC. Verapamil was resuspended with water and stored at −20ºC. Rabbit anti-Top1 antibody was purchased from TopoGEN, Inc. (Columbus, OH, USA), and mouse anti-DNA topoisomerase II α (Top2α) antibody was purchased from Medical & Biological Laboratories Co., Ltd. (Nagoya, Japan).

### Establishment of highly camptothecin-resistant colon cancer cell sublines

**Figure 1:**
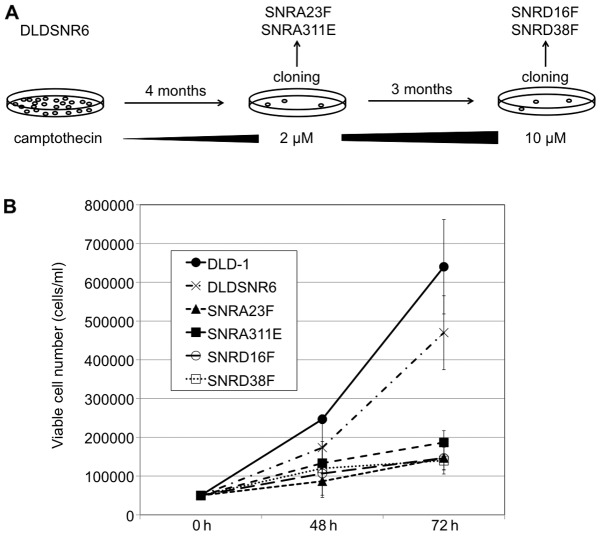
Establishment of highly camptothecin-resistant DLD-1 sublines. (A) Schema of the generation of highly camptothecin-resistant DLD-1 cell subclones. (B) Cell growth assay. Each subline was cultured, and the viable cell number was counted by a trypan blue dye exclusion test. The values are expressed as means ± standard deviation (SD). The experiment was performed in 3 different cultures.

The DLD-1 human colon cancer cell line was provided by the Cell Resource Center for Biochemical Research of Tohoku University (Sendai, Japan). We previously established the DLDSNR6 cell line from parental DLD-1 cells through the continuous exposure to stepwise increases in SN-38 concentrations (7). In this study, DLDSNR6 cells were exposed to stepwise increases in camptothecin concentrations (up to 2 μM) over a period of 4 months and then SNRA23F and SNRA311E sublines were established by the limiting dilution technique. The camptothecin-resistant cell pool was again exposed to camptothecin with concentrations up to 10 μM for 3 months, and SNRD16F and SNRD38F sublines were obtained (Fig. 1A). These cell lines were cultured at 37ºC in RPMI-1640 medium (Life Technologies Japan, Tokyo, Japan) that was supplemented with 10% fetal bovine serum (Thermo Fisher Scientific K.K., Yokohama, Japan) and Antibiotic-Antimycotic Mixed Solution (Nacalai Tesque, Inc., Kyoto, Japan) under a humidified atmosphere containing 5% CO_2_.

### Cell growth, viability and cytotoxicity assays

DLD-1, DLDSNR6, SNRA23F, SNRA311E, SNRD16F and SNRD38F cells (5.0×10^5^/ml) were cultured in 6-cm culture dishes for 48–72 h. The number of viable cells was counted by the trypan blue dye exclusion method with a hemocytometer. The cytotoxicity of Top1-targeting drugs was measured by an MTS assay (Cell Titer 96 Aqueous One Solution Cell Proliferation Assay; Promega, San Luis Obispo, CA, USA) with minor technical modifications. Three to ten thousand cells were incubated in a 96-well tissue culture plate for 96 h in the presence of the indicated concentrations of camptothecin, SN-38, and J-107088, after which the assay was performed according to the manufacturer's instructions. All the experiments were performed in triplicate.

### Detection of Top1 mutations

Total RNA was extracted from each cell line and reverse transcription was performed. The full-length Top1 cDNA was amplified by polymerase chain reaction (PCR), and the resulting fragments were inserted into the cloning vector. The entire Top1 open reading frame was sequenced with the BigDye Terminator Version 3.1 Cycle Sequencing kit (Life Technologies Japan) and the ABI 3700 DNA Analyzer (7,10).

### Preparation of protein samples and immunoblotting analysis

Crude cell extracts were prepared by suspending the cells in radioimmunoprecipitation assay lysis buffer containing 1 μM phenylmethylsulfonyl fluoride, and the protein concentrations of each sample were measured by the Bradford method. Protein samples were separated by 7.5% sodium dodecyl sulfate-polyacrylamide gel electrophoresis and immunoblotting was performed with antibodies for Top1 and Top2α.

### Top1-mediated DNA relaxation assay

The Top1 catalytic activities of the nuclear extracts from each cell line were determined by measuring the relaxation of the supercoiled pHOT1 plasmid (TopoGEN, Inc.), which contained a Top1-cleavage site that was derived from the tetrahymena ribosomal gene repeat (11). The supercoiled pHOT1 plasmid (0.25 μg) was incubated with the indicated amounts of nuclear extracts in 10 mM Tris-HCl (pH 7.5), 150 mM NaCl, and 1 mM ethylenediaminetetraacetic acid (EDTA) at 37ºC for 60 min in a final volume of 20 μl (7). The reaction was terminated by the addition of 5 μl of 0.05% sodium dodecyl sulfate and the samples were loaded onto 1% agarose gels. After electrophoresis, the gels were stained with Tris-borate EDTA buffer (89 mM Tris-borate, 2 mM EDTA, pH 8.0) containing 0.5 μg/ml ethidium bromide and visualized by transillumination with UV light. Relaxation activity was identified by the disappearance of the supercoiled DNA.

### Quantitative reverse transcription (qRT)-PCR array analysis of ATP-binding cassette transporters

To assess the relative expression of ATP-binding cassette transporters in each cell line, we used the TaqMan™ Array Gene Signature 96-well plates (human ABC transporters; Life Technologies Japan). After 2 months of passage in drug-free medium, the cells were harvested, and the total RNA was extracted with ISOGEN reagent (Nippon Gene, Tokyo, Japan). The analysis was performed according to the manufacturer's directions with the Applied Biosystems 7500 Real-Time PCR system (Life Technologies Japan). As a measure of the relative levels of expression between the parental DLD-1 and the resistant cell lines, ΔΔCt values were calculated and converted to fold-change values (2^−ΔΔCt^).

### Flow cytometry

The effects of J-107088 on cell cycle distribution in each cell line were determined with propidium iodide staining and analyzed with flow cytometry. Each cell line was either treated with vehicle alone [Me_2_SO (0.2%)], J-107088 (5 μM), J-107088 (5 μM) plus verapamil (10 μM), or J-107088 (5 μM) plus Ko143 (0.3 μM) for 48 h. Cells were harvested, fixed in 70% precooled ethanol, and incubated in phosphate-buffered saline containing 10 μg/ml propidium iodide and 10 μg/ml RNase for 30 min at room temperature. The fluorescence (excitation at 488 nm and emission at 585/42 nm) of 2×10^4^ cells from each sample was analyzed with FACSCalibur (Becton-Dickinson, San Jose, CA, USA) flow cytometry, and the cell population at each cell cycle phase was determined with ModiFit software (Becton-Dickinson).

## Results

### Establishment of highly camptothecin-resistant colon cancer cells

**Figure 2:**
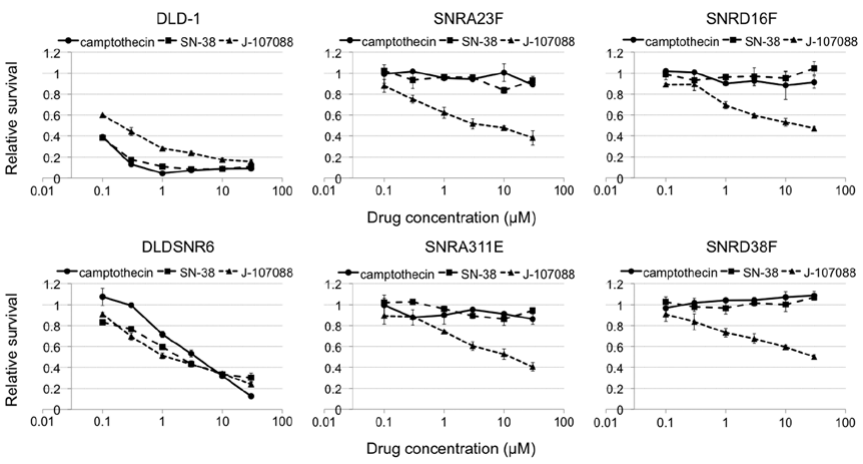
Cytotoxicity of topoisomerase I-targeting drugs. Cells were cultured for 96 h in the presence of the indicated camptothecin, SN-38 and J-107088 concentrations. Cell viability was measured by an MTS assay. The values are expressed as means ± SD (n=3).

In this study, we established the SNRA23F, SNRA311E, SNRD16F and SNRD38F sublines, which were highly camptothecin-resistant cell lines (Fig. 1A). These cells exhibited markedly retarded growth compared with that of parental DLD-1 and DLDSNR6 cells (Fig. 1B). Observations of the cells stained with 4′-6-diamidino-phenylindole under a fluorescence microscope revealed no signs of apoptotic cell death in the resistant cell lines (data not shown). The newly established sublines grew in the presence of 30 μM of camptothecin. In addition, these cells were resistant to the indolocarbazole derivative, J-107088 (Fig. 2).

### Three missense mutations of the Top1 gene in resistant cell lines

**Figure 3:**
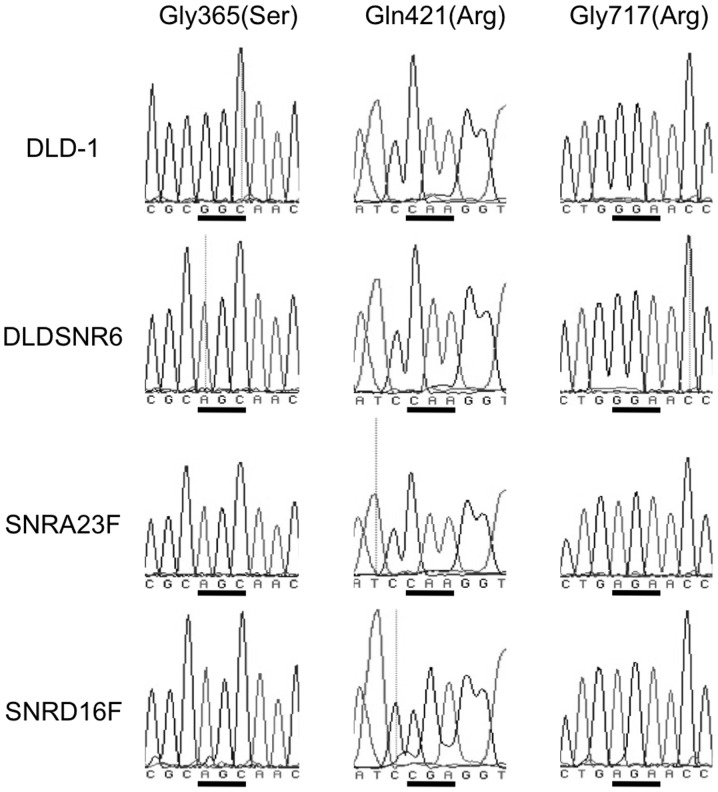
Topoisomerase I (Top1) mutations in highly camptothecin-resistant DLD-1 sublines. Full-length Top1 cDNA was amplified by reverse transcription-polymerase chain reactions, from RNA that was extracted from each clone and sequenced. The SNRA311E cells had the same missense mutations as the SNRA23F cells (Gly365Ser and Gly717Arg). The SNRD38F cells had the same mutations as the SNRD16F cells (Gly365Ser, Gly717Arg and Gln421Arg).

We previously identified a Top1 missense mutation in DLDSNR6 cells at codon 365, which resulted in the amino acid alteration of glycine (GGC) to serine (AGC) (7). In addition, the parental DLD-1 cells had a heterozygous Top1 missense mutation resulting in a Met675Ile alteration (12,13). The highly camptothecin-resistant cells (SNRA23F, SNRA311E, SNRD16F and SNRD38F) harbored a Top1 missense mutation at codon 717, which resulted in a glycine (GGA) to arginine (AGA) substitution. Furthermore, the SNRD16F and SNRD38F cell lines carried a codon 421 mutation that resulted in the substitution from glutamine (CAA) to arginine (CGA) (Fig. 3).

### Top1 protein expression and enzymatic function in resistant cell lines

**Figure 4:**
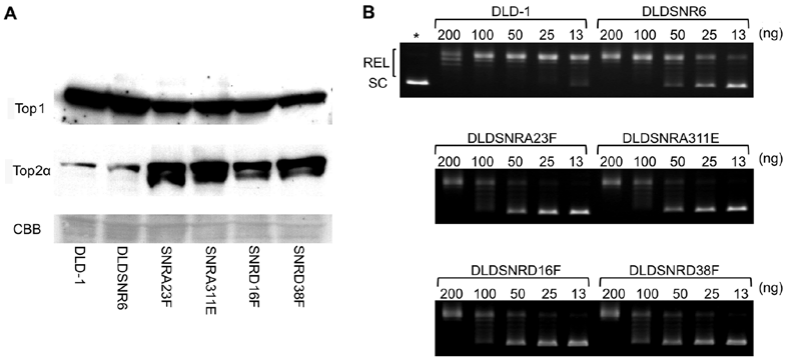
Characterization of the highly camptothecin-resistant cell lines. (A) Topoisomerase I (Top1) and topoisomerase 2α (Top2α) protein expression in resistant cell lines evaluated by western blotting. Coomassie brilliant blue (CBB) staining demonstrated equal protein loading. (B) Top1-mediated plasmid relaxation. Supercoiled pHOT1 plasmid was incubated for 60 min with the indicated amounts of nuclear extracts from DLD-1 and camptothecin-resistant sublines. Asterisk, sample without nuclear extract. SC, supercoiled DNA. REL, relaxed DNA.

There were slightly lower levels of Top1 protein expression in highly camptothecin-resistant cell lines compared with those of the parental DLD-1 and DLDSNR6 cells, whereas the levels of Top2 protein expression were increased in these sublines (Fig. 4A). The DNA relaxation assay revealed that Top1 activity was markedly reduced to approximately one-eighth in highly camptothecin-resistant cell lines compared with that in DLD-1 cells (Fig. 4B).

### Expression of ATP-binding cassette transporters

The levels of mRNA expression of the ATP-binding cassette transporters ABCB1 [multidrug resistance protein 1 (MDR1)] and ABCG2 [breast cancer resistance protein (BCRP)] were significantly increased in camptothecin-resistant cell lines, including DLDSNR6. However, the levels of mRNA expression of ABCC2 (multidrug resistance-associated protein 2) were reduced in SNRA23F and SNRA311E cells (Table I).

### Cell cycle analysis of J-107088-treated cells

**Figure 5:**
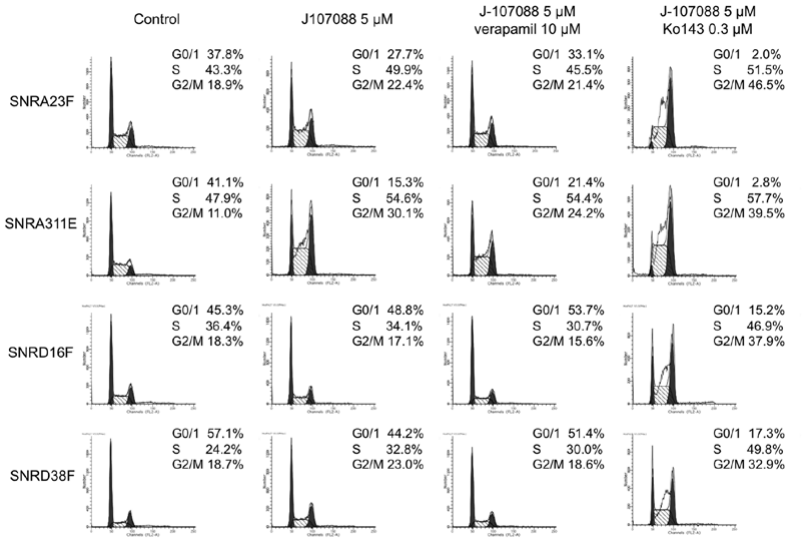
Cell cycle analysis of drug-treated cells. DLD-1 and camptothecin-resistant cell lines were either treated with vehicle alone, J-107088 (5 μM), J-107088 (5 μM) + verapamil (10 mM), or J-107088 (5 μM) + Ko143 (0.3 μM) for 48 h. A DNA histogram analysis was performed by flow cytometry. Representative histograms from at least 2 independent experiments are shown.

The flow cytometry analysis of untreated cells revealed that the S-phase fraction was not reduced in highly camptothecin-resistant cells (Fig. 5) compared with parental cells (~24% in DLD-1 cells, ~30% in DLDSNR6 cells, data not shown). When cells were treated with 5 μM of J-107088 for 48 h, accumulation was observed in the late S-G_2_/M phase in SNRA23F and SNRA311E cells, while the cell cycle distribution was not clearly affected in SNRD16F and SNRD38F cells (Fig. 5). Treatment with camptothecin at the higher concentration of 10 μM caused marginal changes in the cell cycle distribution in highly camptothecin-resistant cells (data not shown). The addition of the MDR1 inhibitor, verapamil, to 5 μM of J-107088 did not evidently affect the cell cycle distribution. When cells were coincubated with the BCRP inhibitor, Ko143, and 5 μM of J-107088 for 48 h, a marked accumulation in late S to G_2_/M phase was observed in all highly camptothecin-resistant cells, while the G_0_/_1_ population was still observed in SNRD16F and SNRD38F cells (Fig. 5).

## Discussion

In the present study, we established highly camptothecin-resistant colon cancer cell lines and characterized these cells. First, the highly resistant clones (SNRA23F, SNRA311E, SNRD16F, and SNRD38F) were retarded in growth and showed slightly lower levels of Top1 protein expression (Fig. 1B and 4A). The previously established DLDSNR6 cells exhibited a loss of heterozygosity in the Top1 gene, but these cells did not exhibit significant growth retardation. Toyoda *et al*(14) demonstrated the heterozygous disruption of the Top1 gene in a human pre-B cell line, Nalm-6. The TOP1-heterozygous Nalm-6 cells exhibited ~70% protein expression levels of Top1, but they showed no significant differences in the growth rate compared with that of the parental cells. In our highly camptothecin-resistant cells, Top1 enzymatic activity levels were reduced to approximately one-eighth mainly due to the new missense mutations (Fig. 4B). The TOP1 wild-type allele was not expressed in our cells. Top1 knockdown that was conducted with small interfering RNA to the level of ~10–20% in cancer cell lines caused genomic instability and replication defects (15). Minor deficits in Top1 activity may not affect the cell growth rate. Human Top2α, which can relax the positively supercoiled DNA, has been shown to partially compensate for Top1 activity (16). Our newly established resistant cells showed high levels of expression of Top2α (Fig. 4A). The increased levels of Top2α expression in these cells but not in DLDSNR6 cells may suggest that a substantial reduction of Top1 activity levels occurred only in highly resistant cells.

Our highly camptothecin-resistant clones grew in the presence of 30 μM of camptothecin (Fig. 2). These clones harbored two or three missense mutations in the Top1 gene. The X-ray structure of human Top1 has been determined (17,18), and it shows that the cap region (residues 215–433) is sterically close to the catalytic Tyr723. When Top1 clamps double-stranded DNA, some loop regions focus on one side of the DNA (17,19). It has been proposed that amino acids 360–370 of Top1 form a loop region, which contacts other loop regions (residues 417–423, 496–505 and 529–538) to create a salt bridge and two non-covalent bonds between the cap region and the bottom lobe of the enzyme (19). Several structural models have demonstrated that camptothecin derivatives mimic a DNA pair and inhibit the DNA religation activity of Top1 by stabilizing the covalent Top1-DNA complexes (1,20). Moreover, structural models have indicated that camptothecin derivatives interact with Arg364, Asp533 and Asn722 of Top1 (17,20). The newly identified Top1 mutations in this study were positioned in or near the residues that have been shown to be important for enzyme-DNA interactions or enzyme-drug interactions.

The camptothecin-resistant cell lines overexpressed MDR1 and BCRP (Table I). The overexpression of ATP-binding cassette transporters is often responsible for the cellular resistance to anticancer drugs. Camptothecin derivatives and indolocarbazole Top1 inhibitors have been demonstrated to be effectively effluxed by BCRP, while camptothecin is a relatively poor substrate for MDR1 (21–23). Verapamil and Ko143 could not enhance the effects of camptothecin on the highly resistant cells in terms of the growth rate or cell cycle progression (data not shown). The higher concentration of camptothecin plus verapamil or Ko143 could not trap covalent enzyme-DNA complexes by a band depletion assay in these cells (data not shown). The established cells in this study were ~15 to 150-fold resistant to the indolocarbazole derivative, J-107088 (Fig. 2). The exposure of cells to 5 μM of J-107088 plus 0.3 μM Ko143 caused the accumulation of SNRA23F and SNRA311E cells in the late S to G_2_/M phase. In the SNRD16F and SNRD38 cells, this combined exposure caused accumulation in the late S-G_2_/M phase, but the G0/1 population remained in these cells (Fig. 5). These data suggested that J-107088 effectively caused DNA damage in SNRA23F and SNRA311E cells compared with SNRD16F and SNRD38F cells, although the SNRA23 cells expressed more BCRP mRNA than SNRD38F. A Top1Gln412Arg mutation may confer further resistance to this indolocarbazole derivative. In the cytotoxicity assay, the SNRD16F and SNRD38F cells were slightly more resistant to J-107088 at higher concentrations compared with SNRA23F and SNRA311E cells (Fig. 2).

The DLDSNR6 cells have shown a loss of heterozygosity in the TOP1 gene and exhibited genomic instability due to homozygous mutations in the hMSH6 gene (7). This background enabled us to establish the highly camptothecin-resistant cell lines that had three mutations in one allele of the TOP1 gene. To the best of our knowledge, such cell lines have not previously been reported. The Top1Gly717 mutation has been reported in camptothecin-resistant ovarian cancer cells (24). A mutation corresponding to human Top1Glu421 was previously identified in camptothecin-producing plants, but it has not been identified in mammalian cells (8). The camptothecin producing plants have three mutations in the TOP1 gene and mutations in residues sterically near the catalytic tyrosine in addition to the Glu421 mutation. Our newly established cell lines may be useful for understanding enzyme-drug interactions and the molecular evolution of drug resistance.

## Figures and Tables

**Table I:** qRT-PCR array analysis of ATP-binding cassette transporters.

Gene	DLD-1	DLDSNR6	SNRA23F	SNRA311E	SNRD16F	SNRD38F
ABCA2	1.000	0.871	0.871	0.824	1.602	0.807
ABCB1	1.000	6.774	11.314	17.268	12.295	7.945
ABCB4	1.000	2.028	2.990	2.621	1.505	1.117
ABCC1	1.000	1.141	1.087	0.914	0.927	0.883
ABCC2	1.000	0.486	0.156	0.071	0.620	0.374
ABCC3	1.000	0.753	0.901	0.883	0.835	0.901
ABCC4	1.000	1.050	0.801	0.889	0.914	1.007
ABCC5	1.000	1.035	1.173	1.240	1.000	0.841
ABCC6	1.000	1.338	2.868	3.364	3.227	2.445
ABCC10	1.000	1.548	2.085	2.532	1.753	2.129
ABCC11	1.000	0.940	0.274	0.406	0.339	0.412
ABCG2	1.000	3.031	6.105	2.497	9.580	4.199

qRT-PCR, quantitative reverse transcription-polymerase chain reaction. Relative expression of ATP-binding cassette transporters between the parental DLD-1 and the resistant sublines. ΔΔCt values were converted to fold-change.
